# Animation-Based Lectures in Renal Physiology: Transcendence into Metacognition

**DOI:** 10.3352/jeehp.2009.6.6

**Published:** 2009-12-20

**Authors:** Satendra Singh, Shikha Gautam

**Affiliations:** Department of Physiology, University College of Medical Sciences, Dilshad Garden, Delhi, India.

Renal physiology has often been the most difficult topic for students to grasp and provides pedagogical challenge to educators. It has an inherent difficulty associated with it (intrinsic cognitive load). This inherent difficulty may not be altered by an instructor by didactic lecture or by static slides without animation (extraneous cognitive load) and it then becomes difficult for the student to understand core concepts in renal physiology like renal regulation of acid-base balance through ion exchange (eSlide).

The first example is that of typical conventional diagram provided in almost all physiology text books. To learn from these diagrams learners must split their attention between various arrows to understand and grasp ion exchange. This is a typical example of Split-attention effect (extraneous cognitive load) and eludes elucidation as it has to be decoded mentally to which many learners may not be adept at and also our working memory capacity is limited. The later slides depicting "Integrated example" enhances learning because embellished with animations and displayed sequentially, it guides the learner's attention through the worked example.

The linguistic invention-animation-based lectures (ABL) as a protologism was first used by us in 2009. Majority of the students agreed that ABL helped to sustain interest, visualize concepts better, remembering facts, applying knowledge and understanding better different aspects of physiology teaching (including renal physiology) [1].

Students dislike both transparencies and static PowerPoint slides for the monotony and lack of interest it generates among both the teacher and student. There is richness that occurs while incorporating multisensory modalities as part of ABL [2]. It is aptly considered extension of Gardner's Multiple Intelligences theory in education [3].

According to a cognitive theory of multimedia learning, active learning occurs when a learner engages in the cognitive process of *selecting* relevant words and images, *organizing* words and images into coherent verbal and visual models, and *integrating* the corresponding components of the verbal and visual models [4].

ABL, therefore, as a pedagogical tool in renal physiology is a metacognitive strategy that assists learners and minimizes working memory load and promotes meaningful learning. More ABL as instructional design decrease extraneous cognitive load during learning, and refocus that learner's attention toward germane materials, increasing germane (schema related) cognitive load. Carefully crafted hypotheses and well-matching experiments will go a long way to providing insight into the true value of animations as pedagogical tools.

## References

[1] Singh S, Singh S, Gautam S (2009). Teaching styles and approaches: medical student's perceptions of Animation-Based Lectures (ABL) as a pedagogical innovation. Pak J Physiol.

[2] Stith BJ (2004). Use of animation in teaching cell biology. Cell Biol Educ.

[3] Singh S, Singh S, Gautam S (2009). Pedagogical effectiveness of ABL in appealing multiple intelligences of our medical students: Gardner's theory revisited. Ind Med Gaz.

[4] Moreno R, Mayer RE, Bourdeau J, Heller R (2000). Meaningful design for meaningful learning: applying cognitive theory to multimedia explanations. Proceedings of World Conference on Educational Multimedia, Hypermedia and Telecommunications.

