# Supplementary material for: Animation-Based Lectures in Renal Physiology: Transcendence into Metacognition
Source: J Educ Eval Health Prof. 2009 Dec 20;6:6. doi: 10.3352/jeehp.2009.6.6 (PMC2796728; doi:10.3352/jeehp.2009.6.6)
Supplement: Supplementary file 1 — Supplemental data [file jeehp-6-6-s001.ppt]

## Slide 1
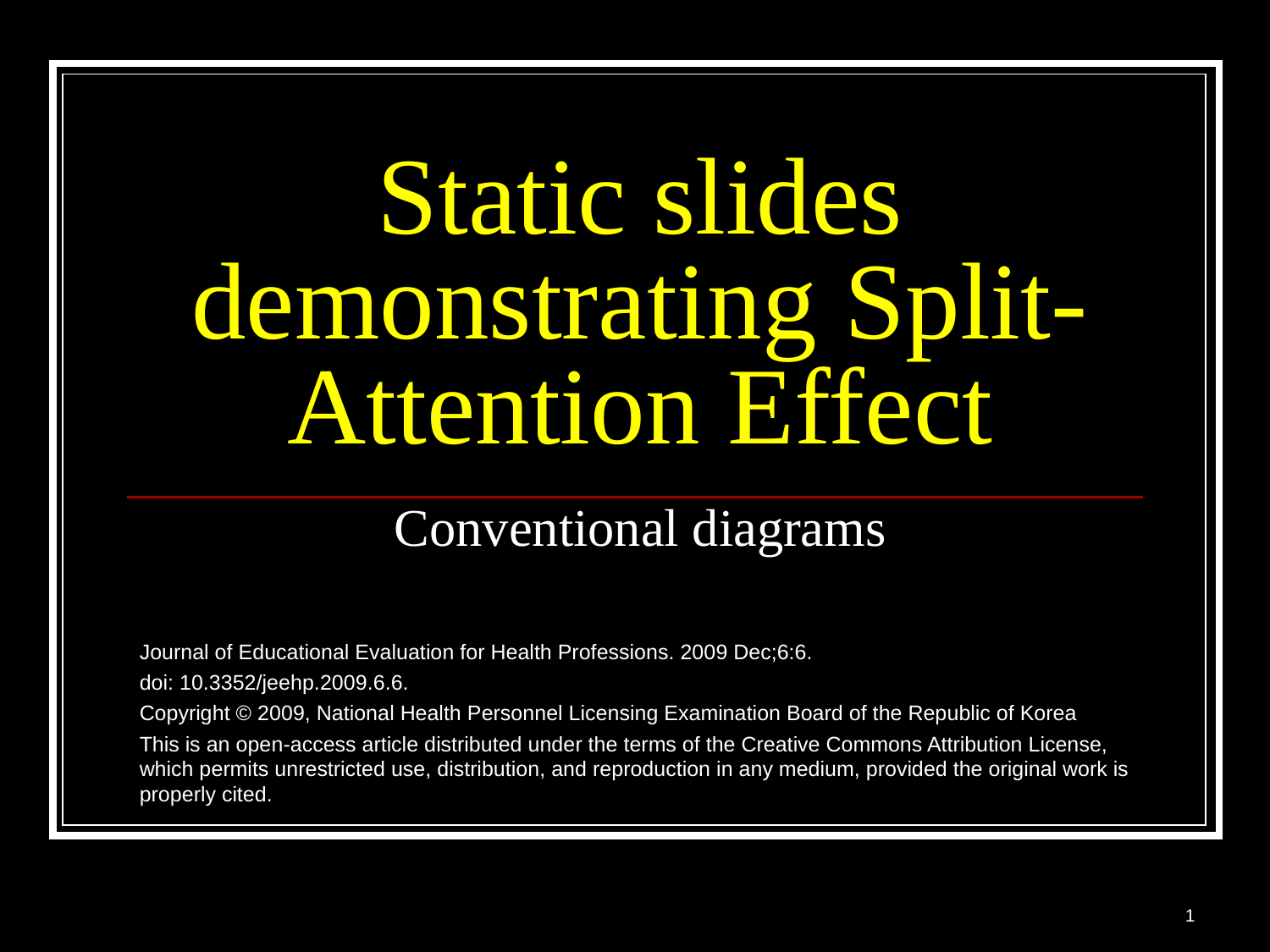

# Static slides demonstrating Split-Attention Effect
Conventional diagrams
Journal of Educational Evaluation for Health Professions. 2009 Dec;6:6.
doi: 10.3352/jeehp.2009.6.6.
Copyright © 2009, National Health Personnel Licensing Examination Board of the Republic of Korea
This is an open-access article distributed under the terms of the Creative Commons Attribution License, which permits unrestricted use, distribution, and reproduction in any medium, provided the original work is properly cited.
<number>

## Slide 2
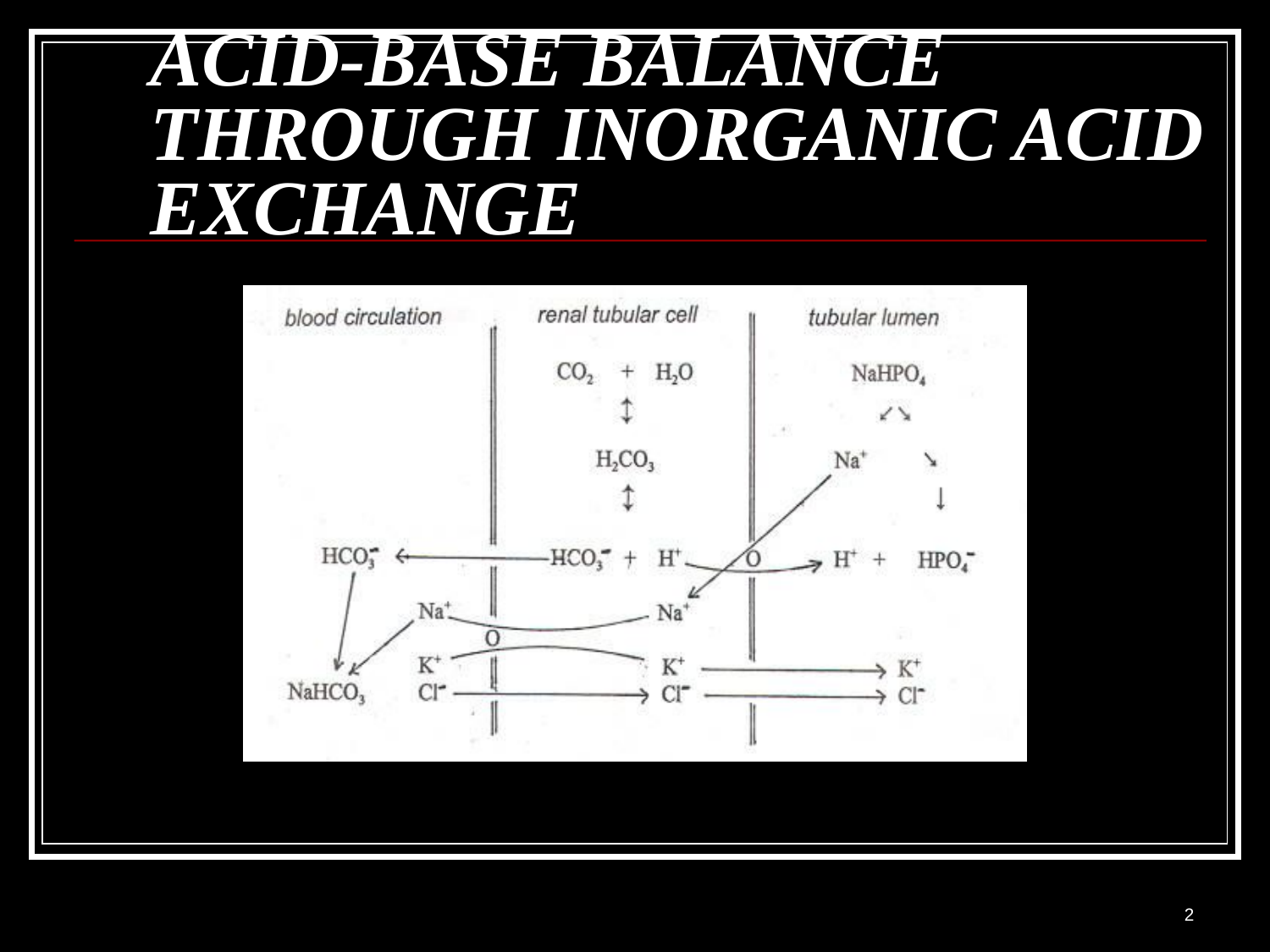

# ACID-BASE BALANCE THROUGH INORGANIC ACID EXCHANGE
<number>

## Slide 3
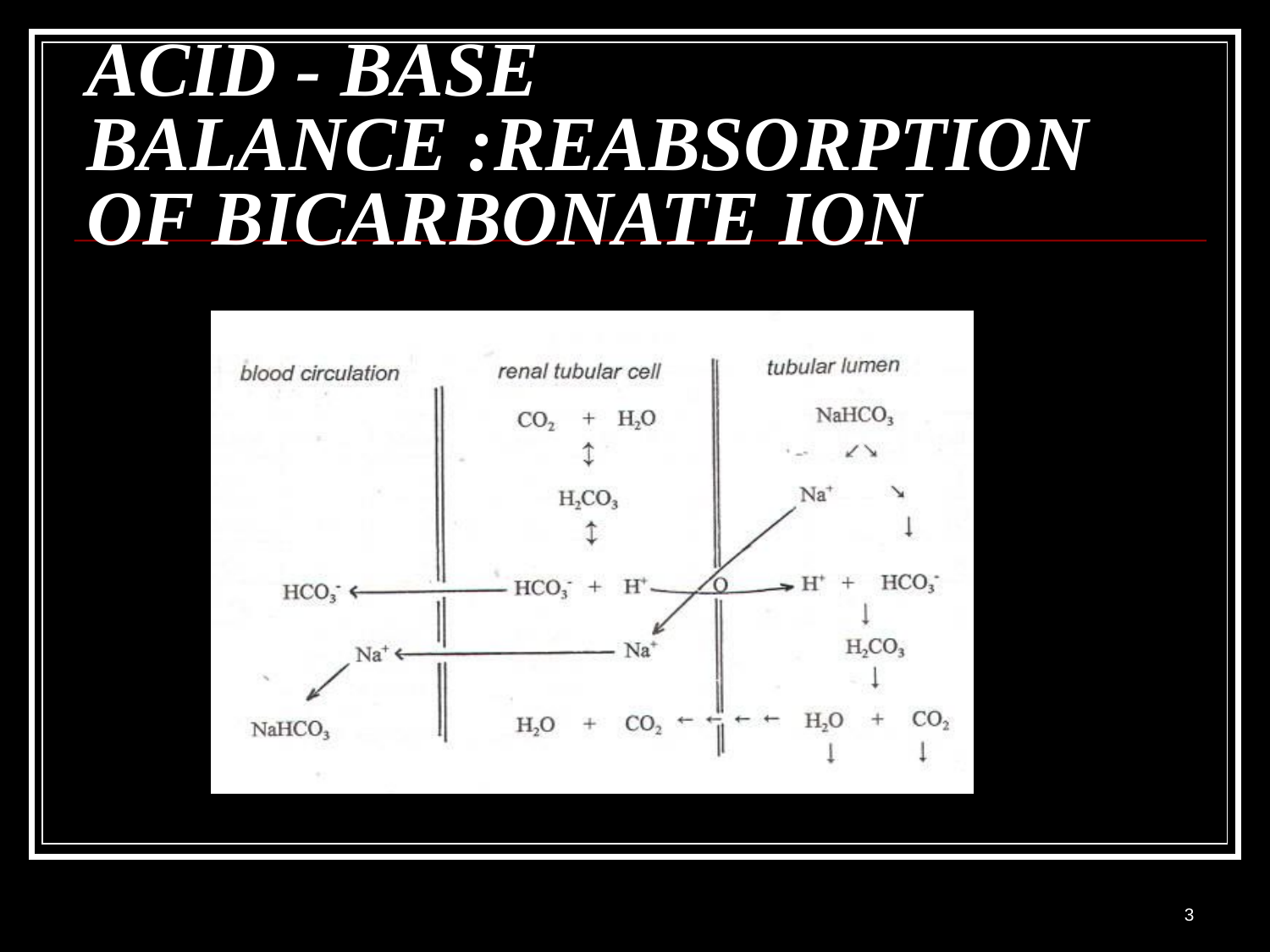

# ACID - BASE BALANCE :REABSORPTION OF BICARBONATE ION
<number>

## Slide 4
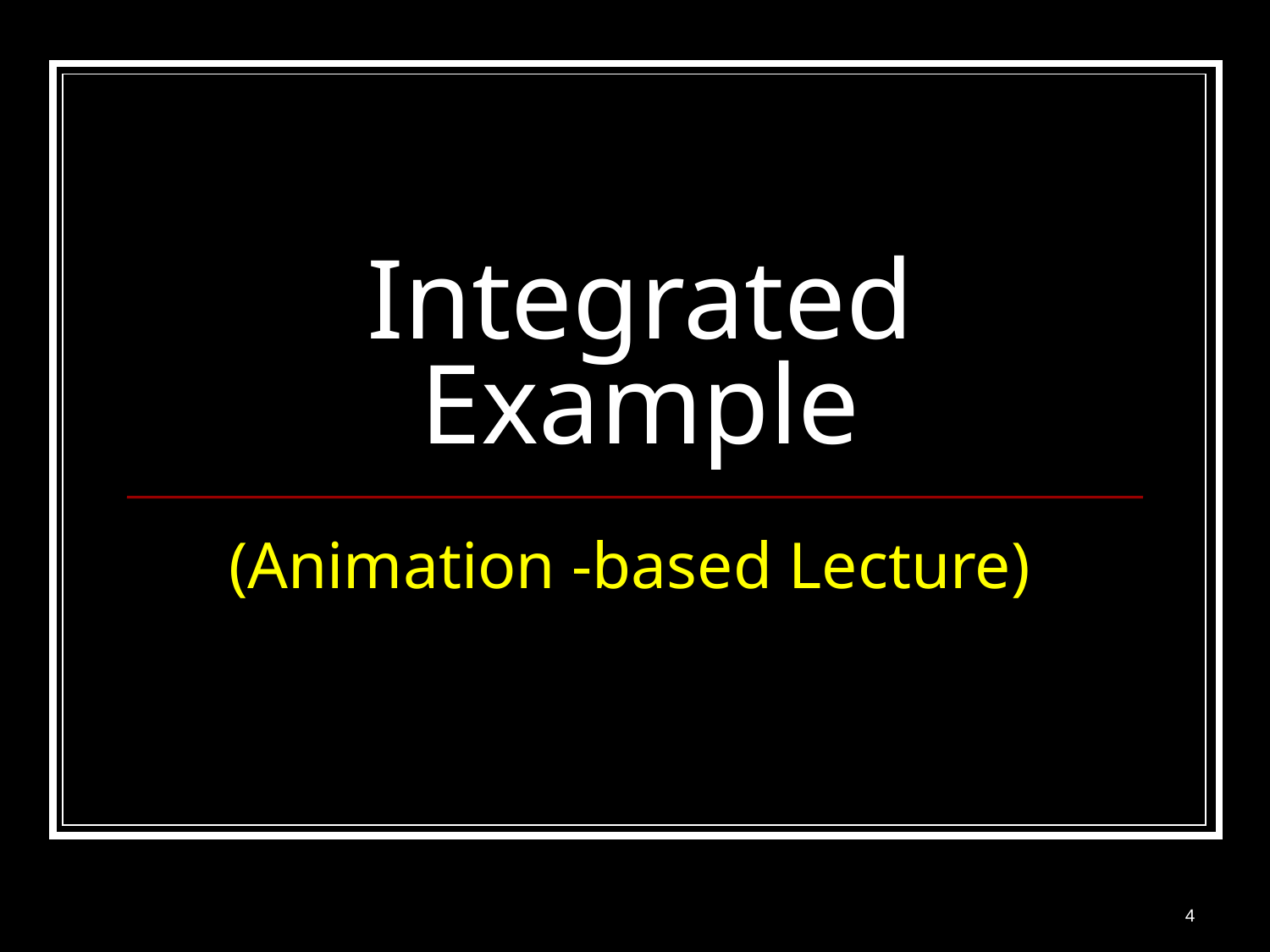

# Integrated Example
(Animation -based Lecture)
<number>

## Slide 5
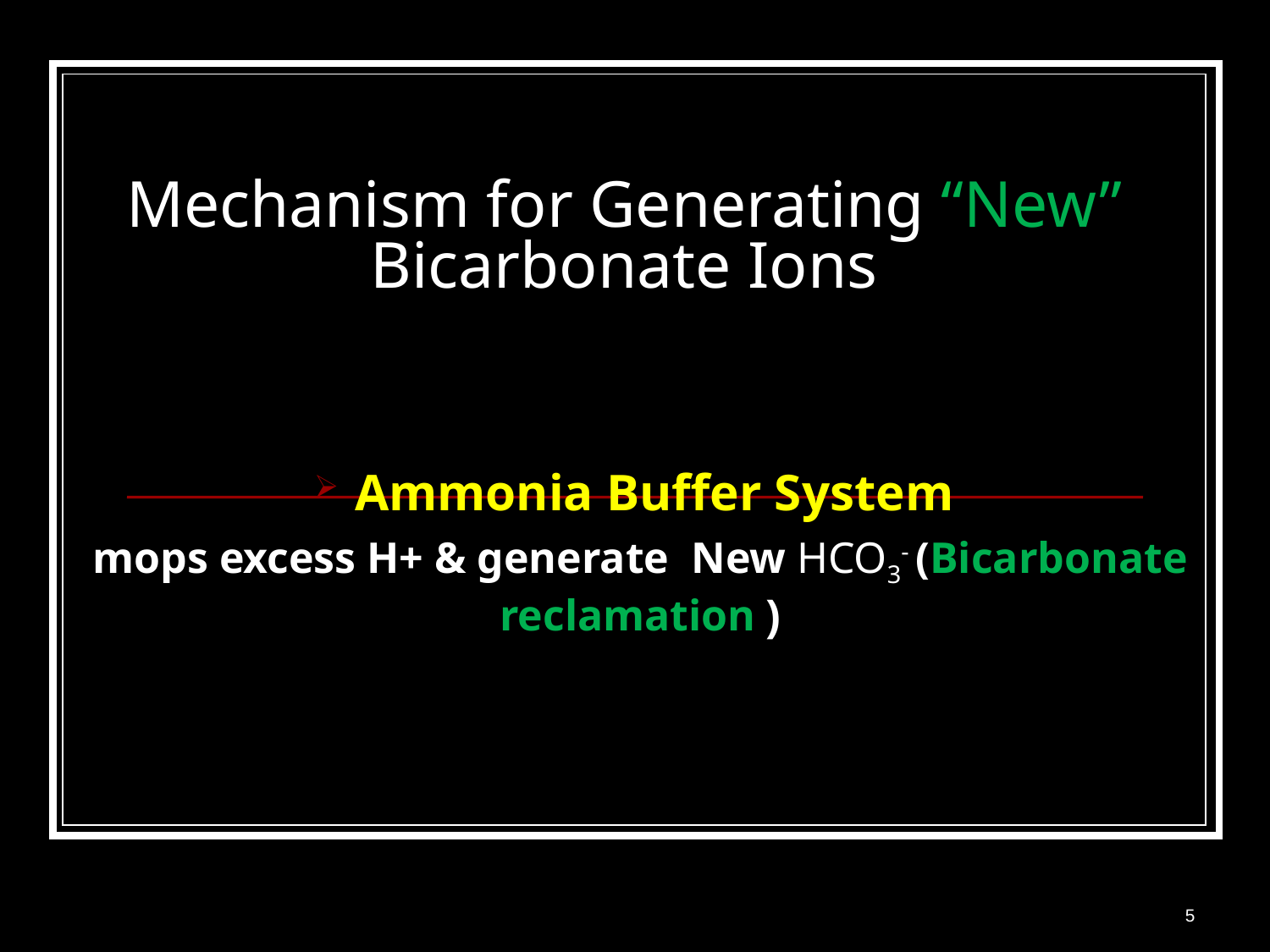

# Mechanism for Generating “New” Bicarbonate Ions
 Ammonia Buffer System
mops excess H+ & generate New HCO3- (Bicarbonate reclamation )
<number>

## Slide 6
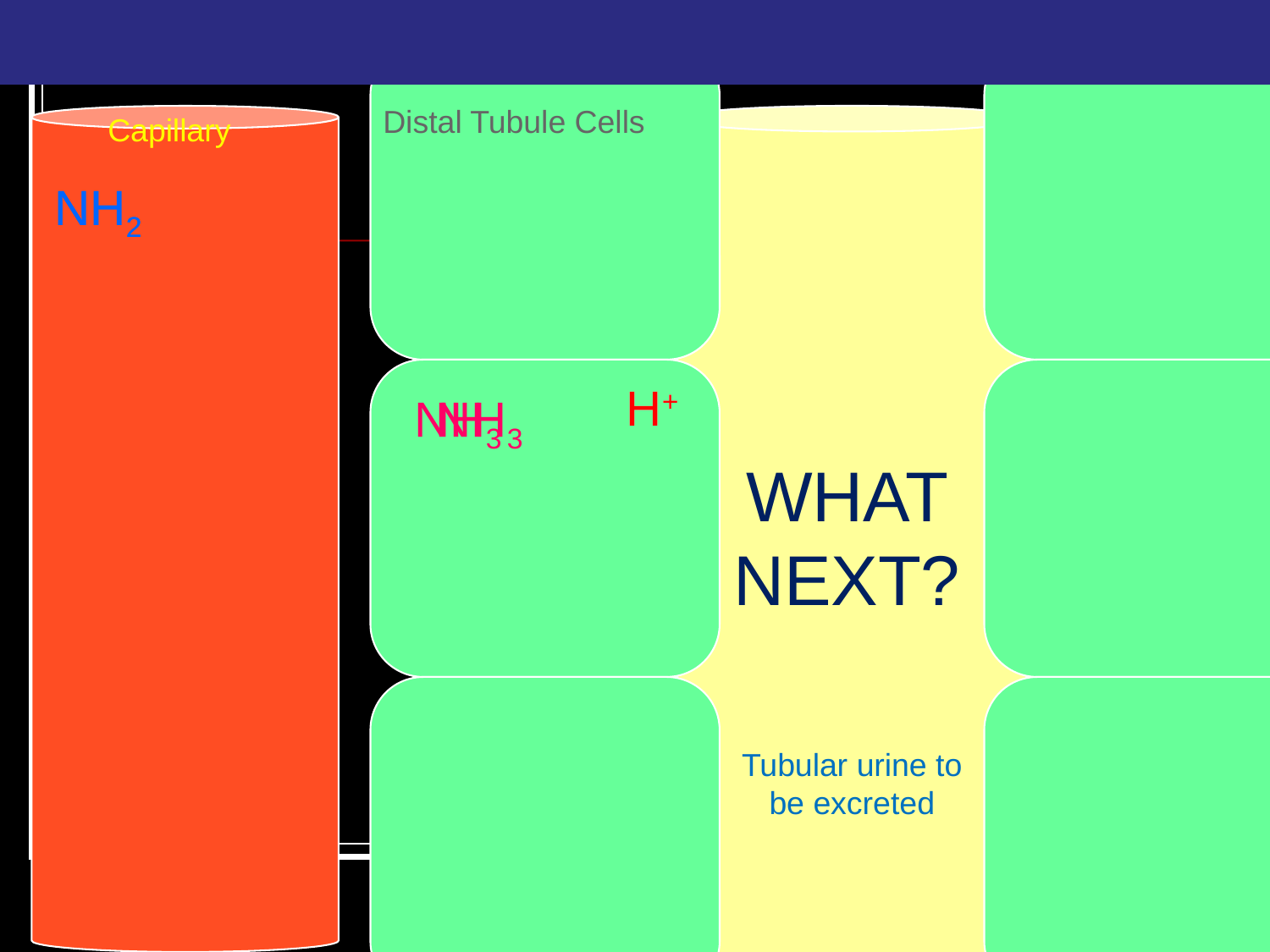

# ACIDIFICATION OF URINE BY EXCRETION OF AMMONIA
Distal Tubule Cells
Capillary
NH2
NH2
H+
H+
NH3
NH3
WHAT NEXT?
Tubular urine to be excreted
<number>

## Slide 7
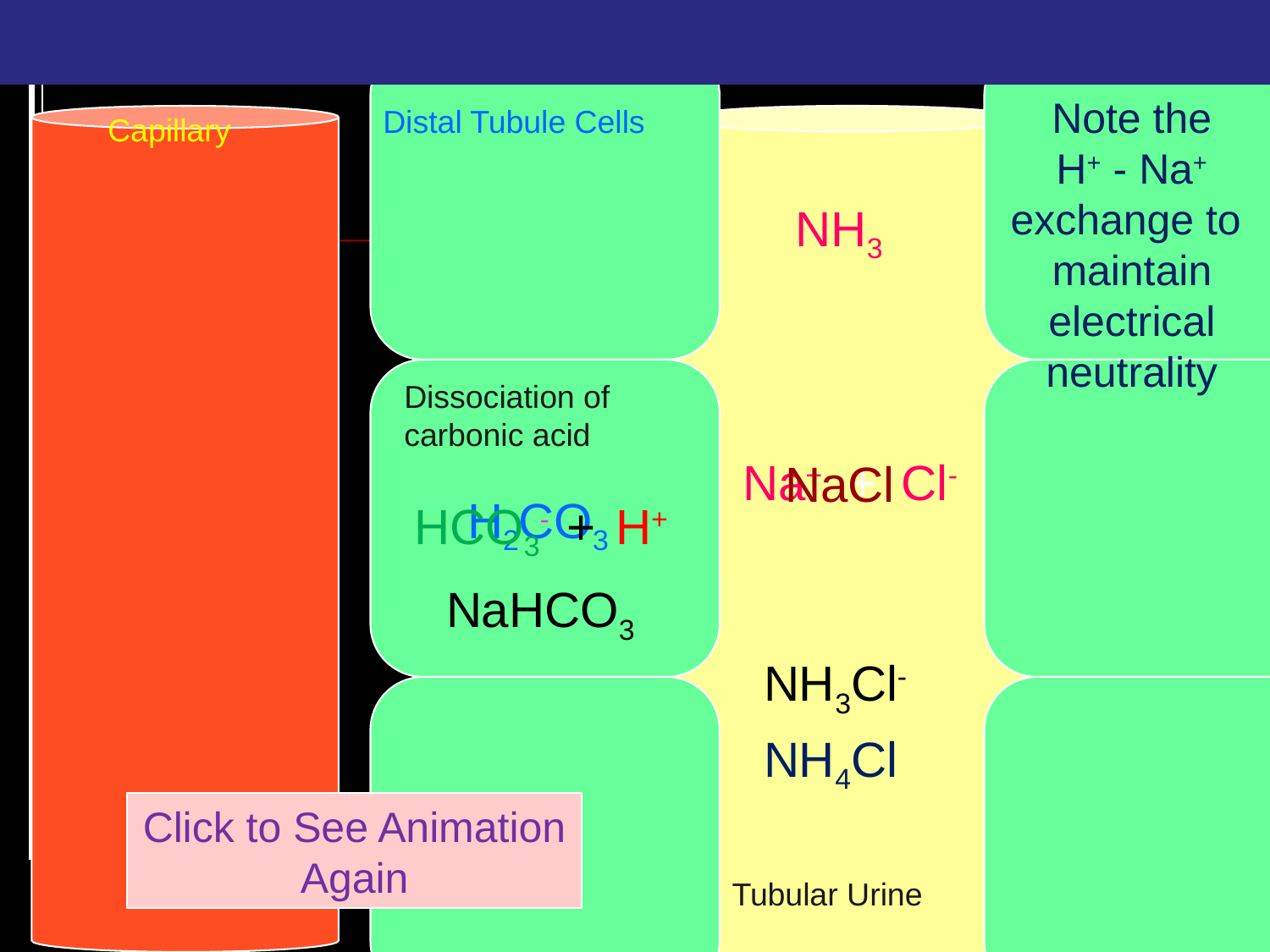

# ACIDIFICATION OF URINE BY EXCRETION OF AMMONIA
Note the
H+ - Na+ exchange to maintain electrical neutrality
Distal Tubule Cells
Capillary
NH3
Dissociation of carbonic acid
Na+
+
Cl-
NaCl
H2CO3
+
HCO3-
H+
NaHCO3
NaHCO3
NH3Cl-
NH4Cl
Click Mouse to Start Animation
Click to See Animation Again
Tubular Urine
<number>

## Slide 8
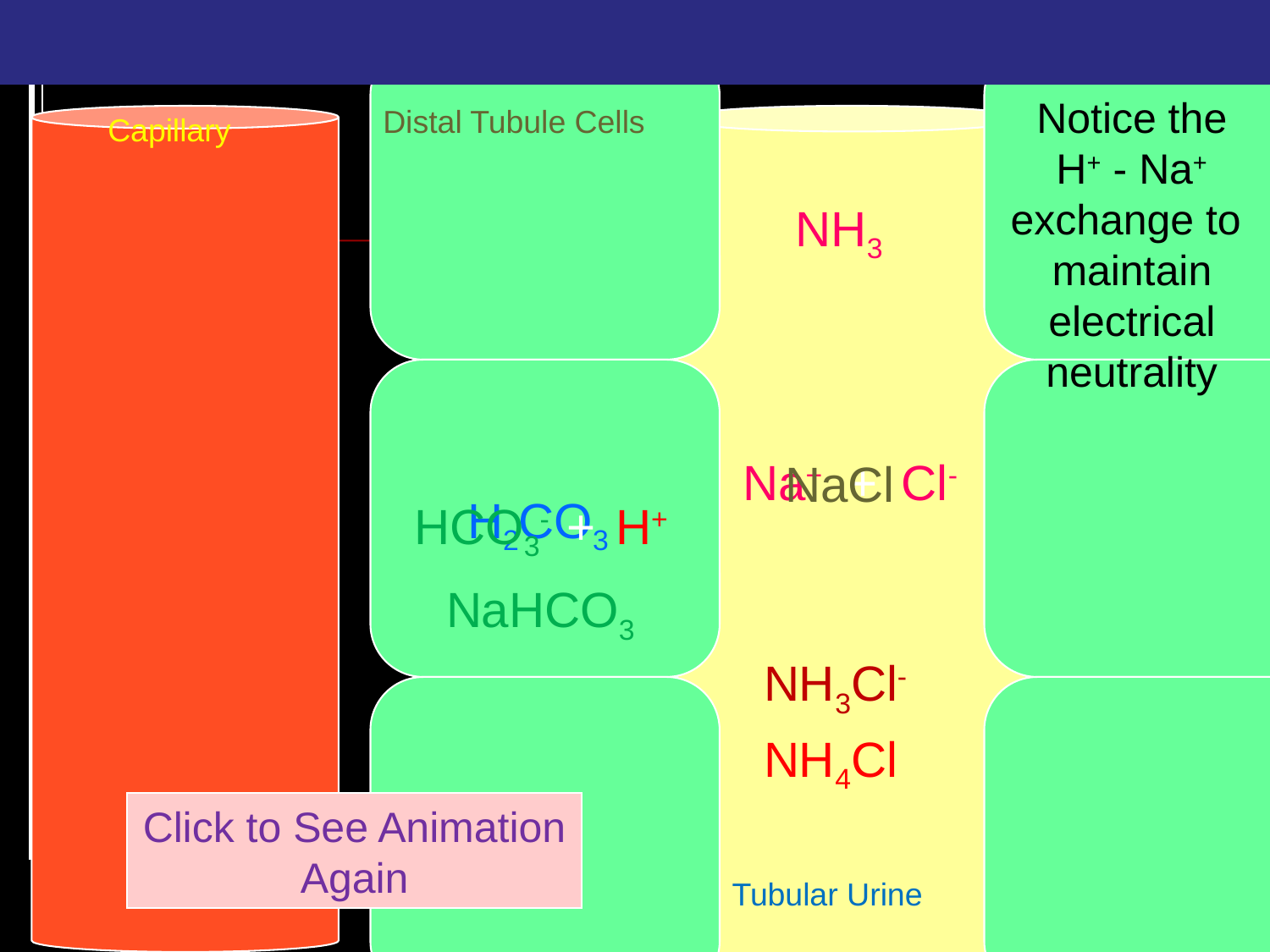

# ACIDIFICATION OF URINE BY EXCRETION OF AMMONIA
Notice the
H+ - Na+ exchange to maintain electrical neutrality
Distal Tubule Cells
Capillary
NH3
Na+
+
Cl-
NaCl
H2CO3
+
HCO3-
H+
NaHCO3
NaHCO3
NH3Cl-
NH4Cl
Click Mouse to Start Animation
Click to See Animation Again
Tubular Urine
<number>

## Slide 9
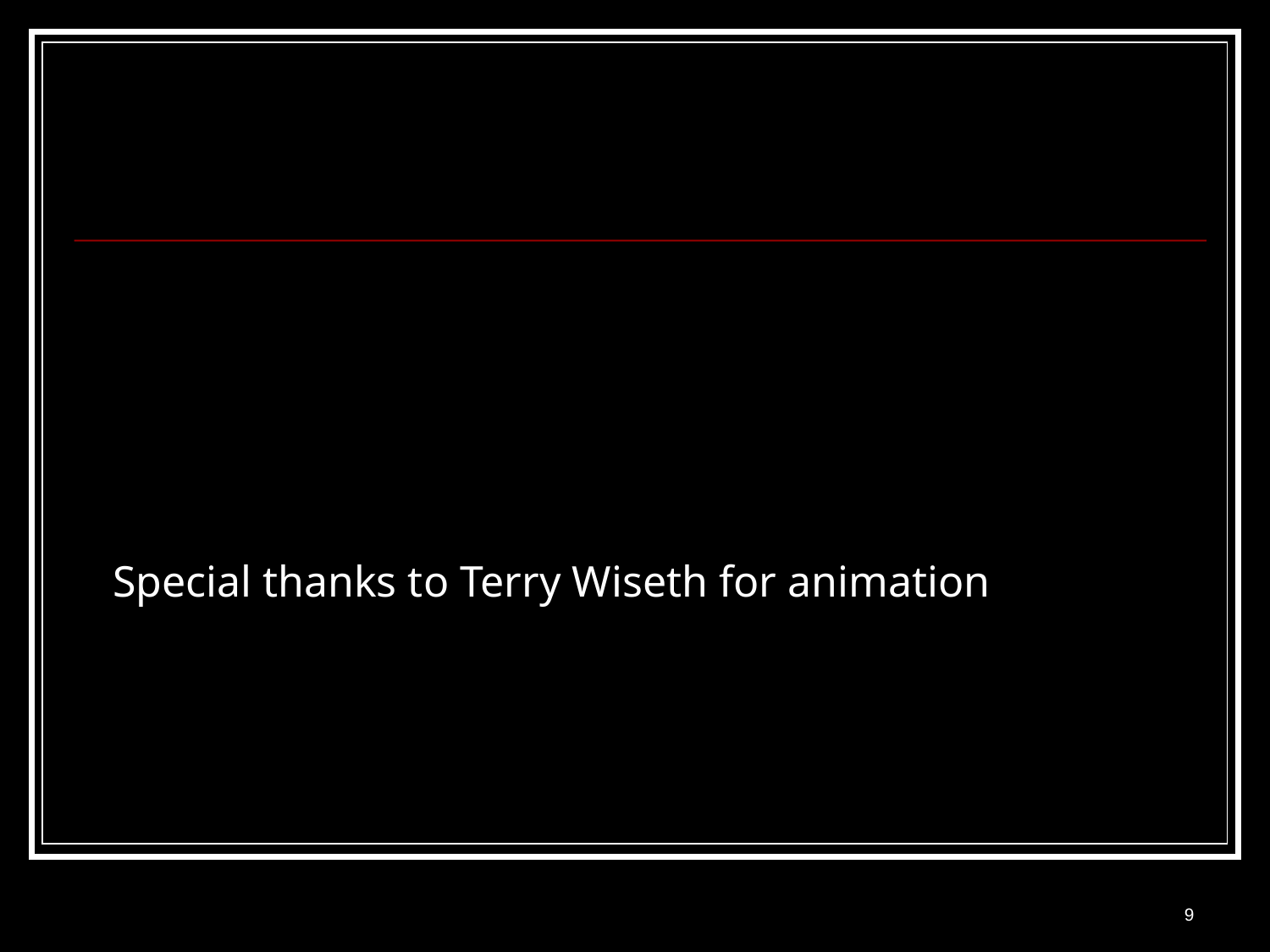

# Special thanks to Terry Wiseth for animation
<number>
